# Anticoagulation quality with warfarin therapy, and associated factors among adult outpatients at public hospitals in nekemte town, western Ethiopia: a retrospective study

**DOI:** 10.3389/fphar.2025.1544957

**Published:** 2025-04-03

**Authors:** Firafan Shuma Teka, Ayana Tadesse Korsa, Habte Gebeyehu Bayisa, Dechasa Befikadu W/Senbat, Refisa Shifera Beyene, Dinka Dugassa Iticha, Birbirsa Sefera Senbeta

**Affiliations:** ^1^ Department of Clinical Pharmacy and Pharmacy Practice, Institute of Health Sciences, Dambi Dollo University, Oromia, Ethiopia; ^2^ Department of Clinical Pharmacy, Institute of Health Sciences, Wallaga University, Oromia, Ethiopia; ^3^ Department of Medical Microbiology, Institute of Health Sciences, Dambi Dollo University, Oromia, Ethiopia; ^4^ Department of Pharmacy, College of Health Science, Mettu University, Oromia, Ethiopia

**Keywords:** anticoagulation quality, associated factors, public hospitals, nekemte town, warfarin

## Abstract

**Background:**

The global prevalence of poor anticoagulation control with warfarin therapy is high. Similarly, the quality of anticoagulation control with warfarin therapy in Ethiopia has been reported to be poor, with a notable paucity of data, especially in the western part of the country.

**Objectives:**

This study aimed to evaluate the anticoagulation quality, and associated factors among adult outpatients on warfarin therapy at Wallaga University Referral Hospital and Nekemte Comprehensive Specialized Hospital, Nekemte town, Western Ethiopia.

**Methods:**

A retrospective study was conducted at public hospitals in Nekemte town from June 1 to 31 July 2023. Data were collected by reviewing patients’ medical charts using a systematic random sampling technique. Time in the therapeutic range was determined using the Rosendaal method. The collected data were entered into EpiData version 4.6.0 and then exported to SPSS version 27.0 for analysis. Bivariable and multivariable logistic regression analyses were performed to identify significant associations. In the multivariable analysis, statistical significance was declared at a p-value of less than 0.05.

**Results:**

A total of 402 patient medical charts with warfarin indications were reviewed. The mean age of the study participants was 38.9 ± 17.9 years, and 271 (67.4%) were female. Good warfarin anticoagulation quality was observed in 36 (9%) of the patients. Aspirin use (AOR = 2.685; CI: 0.872–10.277; p-value = 0.002) and congestive heart failure (AOR = 4.392; CI: 1.028–18.768; p-value = 0.046) were identified as independent predictors of poor anticoagulation quality.

**Conclusion:**

Aspirin use and congestive heart failure were independent predictors of poor anticoagulation quality with warfarin therapy.

## Introduction

Warfarin is a widely used oral anticoagulant for the prevention and treatment of thromboembolic disorders, including deep vein thrombosis (DVT), pulmonary embolism (PE), atrial fibrillation (AF), and mechanical heart valve (MHV) thrombosis (Patel et al., 2023). Despite its efficacy, warfarin has a narrow therapeutic index (Leh-Ching Ng et al., 2019; Alghadeeer et al., 2020; Engell et al., 2021; Hakem et al., 2018; An et al., 2017), requiring frequent monitoring and dose adjustments to maintain the international normalized ratio (INR) within the target range (2.0–3.0 for most indications, and 2.5–3.5 for MHV) (Smith et al., 2021). Time in therapeutic range (TTR) is a key measure of warfarin management quality, with higher TTR associated with better clinical outcomes (Schulman and Crowther, 2020). Warfarin is the most affordable oral anticoagulant (Laäs et al., 2018). Warfarin-treated patients are more closely monitored by using the INR of prothrombin time (PT) with a linearly interpolated TTR (Qiu et al., 2021). TTR indicates the days with INRs of 2.0–3.0 over total day counts (Misleading, 2017), and it is used to measure warfarin anticoagulation quality (Health et al., 2019). To maintain the optimal therapeutic outcome, TTR should be ≥ 65% (Pastori et al., 2015), while the current European Cardiac Society (ECS) guidelines recommend TTR ≥70% (Hindricks et al., 2020). Thus, patients on warfarin therapy have been reported to benefit from a TTR of ≥65% when there is a risk of thromboembolic and bleeding events (Mwita et al., 2018).

Globally, the usage of warfarin therapy is sub-optimal, and only an estimated 15%–44% of patients who are eligible for anticoagulants receive a prescription for warfarin therapy (Health et al., 2019; Mwita et al., 2018). According to data from the United States, for warfarin-treated patients after developing VTE, mean TTR was lower over shorter treatment durations (TTR 30 days vs. TTR 180 days [mean ± SD]: 43.8% ± 33.5% vs. 58.8% ± 23.5%) (Din et al., 2023). Like other anticoagulants, the use of warfarin necessitates careful and routine laboratory monitoring to reduce the risk of bleeding and achieve optimal therapeutic outcomes (Hindricks et al., 2020; Mwita et al., 2018).

According to a 13-year retrospective study published in 2019 in Australia, Indigenous people experienced significantly less time in the therapeutic range compared to non-Indigenous Australians (40% ± 29% vs. 50% ± 31%) (Nguyen et al., 2020). In the real clinical scenario, maintaining adequate anticoagulation control with warfarin therapy has proved challenging due to the complexity of the medication. In line with this, reports from various studies revealed suboptimal anticoagulation with warfarin therapy by documenting low TTRs (<65%) (Mwita et al., 2018; Biedermann, 2017; Gateman et al., 2017; Pokorney et al., 2015; Shehab et al., 2012).

TTR in Western countries with some Eastern or Asian region, ranging from 77% in the Swedish population, 64% in the United States population to 49% and 36% in the Indian (Hakem et al., 2018). In Poland (Sawicka-Powierza et al., 2018), Spain (Sa et al., 2015), and China (Qiu et al., 2021), poor anticoagulation quality with warfarin therapy was reported with low TTR of 55%, 47.3%, and 32%, respectively. In Africa, only 10.4%–32.3% of patients who used warfarin for various indications achieved optimal anticoagulation control (Tadesse et al., 2022). The proportion of patients on VKAs with optimal anticoagulation control in Sub-Saharan Africa (SSA) is 0%–41% (Mwita et al., 2018; Haas et al., 2016; Semakula et al., 2021; Ebrahim et al., 2018).

Despite its well-established efficacy, anticoagulation control with warfarin remains suboptimal globally, with patients in low- and middle-income countries (LMICs) such as Ethiopia being disproportionately affected (Yimer et al., 2021). In Ethiopia, the concern over poor anticoagulation control is magnified by several interrelated factors. First, limited access to regular INR monitoring due to resource constraints is a critical barrier (Alemu et al., 2022). Second, a lack of standardized anticoagulation management services and clinical guidelines tailored to the local context exacerbates the problem (Abegaz et al., 2020). Third, poor patient knowledge regarding anticoagulation therapy and inadequate adherence to medication and dietary recommendations further complicate INR stability (Tadesse et al., 2024). Additionally, the burden of communicable and non-communicable diseases in Ethiopia often leads to polypharmacy, which increases the risk of drug interactions with warfarin (Teklay et al., 2014).

Based on a recent systematic review in Ethiopia, only one-third (32.15%) of patients who used anticoagulants achieved the target INRs within the therapeutic range (Leh-Ching Ng et al., 2019; Alghadeeer et al., 2020) (Kebede and Ketsela, 2022). Moreover, a recent systematic review revealed a TTR range of 13.7%–57.3% to demonstrate poor oral anticoagulation control in patients treated with warfarin therapy in Africa (Tadesse et al., 2022). Similarly in Ethiopia, warfarin anticoagulation control was reportedly poor with TTR ranging from 29% to 47%, which could harm the patients’ intended treatment outcomes (Fenta et al., 2017; Liyew et al., 2021; Yimer et al., 2021; Masresha et al., 2021; Getachew et al., 2023).

Drug-drug interaction (DDI), inter-individual variability, genetic differences, the need for close monitoring, and potential adverse events caused by warfarin therapy limit its quality of use (Asarcıklı et al., 2021). When taken with certain medications or foods, warfarin’s action may be either increased or reduced (Colet et al., 2018). Renal disease, routine use of non-steroidal anti-inflammatory drugs and antiplatelet therapy, and absence of angiotensin receptor blockers are known to affect the anticoagulation quality with warfarin therapy (Sa et al., 2015). Other factors that contribute to poor anticoagulation quality with warfarin therapy are comorbidities such as heart failure, diabetes mellitus, and stroke (Nelson et al., 2013), genetic variation (Alghadeeer et al., 2020), and female gender (Takamoto et al., 2021).

In many western countries, warfarin therapy achieves a mean TTR of approximately 60%–70%, whereas in other regions, including developing countries, TTR remains suboptimal due to various factors such as genetic polymorphisms, drug interactions, and healthcare accessibility (Jones et al., 2022). Given these challenges, understanding the factors affecting TTR and clinical outcomes in our study population is crucial for optimizing anticoagulation therapy. Therefore, this study aimed to evaluate the anticoagulation quality with warfarin therapy, and associated factors among adult outpatients at Wallaga University Referral Hospital (WURH) and Nekemte Comprehensive Specialized Hospital (NCSH), Nekemte Town, Western Ethiopia.

## Methods and materials

### Study setting and period

The study was conducted on adult outpatients receiving warfarin therapy at the chronic care clinics of Wallaga University Referral Hospital (WURH) and Nekemte Comprehensive Specialized Hospital (NCSH), located 330 km west of Addis Ababa, the capital of Ethiopia. According to a health management information system report from 2023, WURH and NCSH have 300 and 208 beds, respectively. Patient medical records of individuals receiving warfarin therapy from 1 April 2021, to 31 March 2023, at the chronic care clinics of WURH and NCSH were retrieved, and data were collected from June 1 to 31 July 2023.

### Study design

A retrospective study was conducted to evaluate anticoagulation quality and associated factors among adult outpatients receiving warfarin therapy at two public hospitals in Nekemte town.

## Populations

### Source populations

All adult patients receiving warfarin therapy at the chronic care clinic of WURH and NCSH.

### Inclusion and exclusion criteria

#### Inclusion criteria

All adult patients (age ≥18 years) receiving warfarin therapy and who had at least two INR readings from 1 April 2021, to 31 March 2023, in the study settings were included.

#### Exclusion criteria

Patients who had been receiving warfarin therapy for less than 1 month, pregnant women, and illegible patient charts were excluded from the study.

### Sample size determination and sampling technique

The sample size required for this study was determined using a single population proportion formula with the following assumptions: a proportion of 0.4724, representing a mean TTR of 47.24% with warfarin therapy (P = 0.4724), based on a previous study conducted in Ethiopia (Masresha et al., 2021), a 95% confidence interval (CI), and a 5% margin of error (d = 0.05).

**FIGURE 1:**
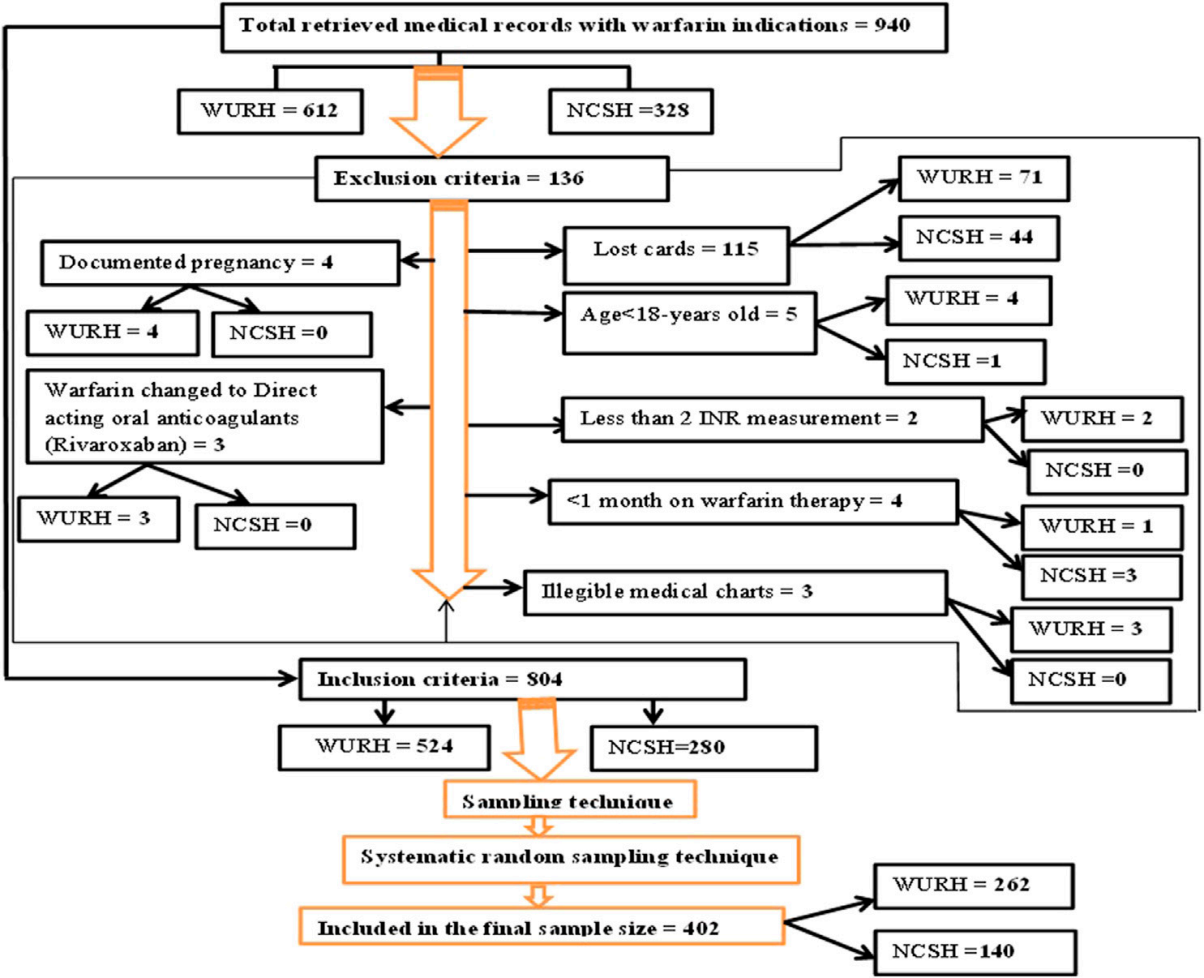
Flow diagram of patient recruitment on warfarin therapy at WURH and NCSH, Nekemte, Western Ethiopia from 1 April 2021-31 March 2023 (N = 402).

A total of 940 patients were on warfarin therapy from 1 April 2021, to 31 March 2023, as recorded in the outpatient registration logbook of WURH and NCSH. Finally, a total of 402 patient medical charts (WURH = 262 and NCSH = 140) were reviewed (Figure 1).

Therefore, the sample size required for this study was calculated based on the above assumption:

$$n=\frac{{\left[z\;\alpha /2\right]}^{2}x\;p\;\left[1-p\right]}{d^{2}}\;n=\frac{{\left(\;1.96\right)}^{2}x\;0.4724\;\left[1-0.4724\right]}{{\left(0.05\right)}^{2}}=383$$

Where n = the sample size, Z = the standard normal distribution with a 95% confidence interval, d = margin of error = 5% (0.05), and p = 0.4724 = proportion of the mean TTR with warfarin therapy.

By adding 10% for the non-response rate (38.3–38), the sample size became 421. Accordingly, the final sample size required for this study was 421.

The sampling interval (K^th^) was determined by dividing the total retrieved medical charts by the calculated sample size as follows:

$$K^{\text{th}}=N/n=940/421=2.2–2$$

Therefore, medical charts of patients with warfarin indications who met the eligibility criteria during the study period were reviewed for every two patient cards using a systematic random sampling technique, where the first two cards were selected by the lottery method.

### Study variables

The quality of anticoagulation with warfarin therapy is the dependent variable, while socio-demographic characteristics, clinical characteristics, and drug-related factors are the independent variables.

### Data collection procedure

#### Data collection tools

Data were collected through patient chart review using a pre-tested data abstraction tool, adopted from prior relevant studies (Fenta et al., 2017; Masresha et al., 2021; Getachew et al., 2023; Of et al., 2016; Teklay et al., 2014; Health, 2016; Zhu et al., 2022) and modified to fit. Since, all patients were treated with the same brand of warfarin, details on the patient socio-demographic characteristics, clinical characteristics, laboratory, and drug-related factors include; age, sex, comorbidity, INR result and frequency of monitoring, indications of warfarin therapy, duration of warfarin use, concurrent medications, and dose of warfarin therapy were collected using a defined data abstraction format. After collecting all this information from medical records, the mean TTR was calculated, and an assessment of INR measures outside the therapeutic range, including the reduction, increase, or omission of warfarin dose, was used to judge the quality of warfarin anticoagulation. INR testing was available at Dambi Dollo University Comprehensive Specialized Hospital during pretest for data collection tools. Morevover, INR testing was available at WURH and NCSH during the study period. Also, this is an exception in Western Ethiopia, made possible through support from Non-Government Organizations (NGOs) and regional health initiatives. The Rosendaal linear interpolation approach (Zhu et al., 2022), which assumes a linear relationship between two INR levels and permits one to assign a specific INR value to each day for each patient, was used to calculate TTR. Patients who enrolled in the study were divided into two groups: The “poor” TTR (TTR < 65%) group and the “good” TTR (TTR ≥ 65%) group. Finally, TTR was calculated using the following formula (Zhu et al., 2022):

$$\%\text{TTR}=\frac{\text{The}\;\text{number}\;\text{of}\;\text{INRs}\;\text{within}\;\text{the}\;\text{target}\;\text{range}\;\text{for}\;\text{all}\;\text{patients}}{\text{Total}\;\text{number}\;\text{of}\;\text{INRs}\;\text{during}\;\text{the}\;\text{selected}\;\text{interval}\;\text{of}\;\text{time}}\;x100$$

#### Recruitment of data collectors

To collect data for this study, a total of four healthcare professionals; 2 Pharmacists and 2 Clinical nurses were recruited. Besides, a 1-day training was given to the data collectors on the overall objective of the study, the data collection format employed, and the data collection process.

### Data quality control

The data collection tool was pre-tested on 21 patients (5% of the sample size); who were on warfarin therapy at Dambi Dollo University Comprehensive Specialized Hospital, Dambi Dollo, Western Ethiopia. All the necessary changes were made accordingly, and hence, the reliability and validity of the tool was maintained. The collected data were regularly checked for completeness and accuracy, and on-site supervision was done daily by the principal investigator. Patient management was conducted by general practitioners and internists at the chronic care clinics of WURH and NCSH.

### Data analysis and interpretations

Data were coded and entered into EpiData version 4.6.0 and then exported for analysis to the statistical package for social science software (SPSS) for Windows version 27.0. The descriptive statistics were computed to describe frequencies, percentages, means, and standard deviations of study variables. Bivariable and multivariable logistic regressions were performed to analyze the association between dependent and independent variables. The model goodness of fit was checked by Hosmer Lemeshow. Variables with a p-value of <0.25 in the bivariable analysis were put into the multivariable logistic regression model to adjust for confounding factors. Before performing the multivariable analysis, a multicollinearity diagnostic was applied to identify whether a significant association occurs between the explanatory variables. In the multivariable analysis, an adjusted odds ratio (AOR) with a 95% confidence interval (CI) was calculated, and the association was declared statistically significant at a p-value less than 0.05 (p < 0.05). Finally, the result was presented in the texts, tables, figures, and graphs form.

### Operational definitions and definitions of terms

#### Comorbidity

Any medical conditions co-exist with indications of warfarin therapy such as deep vein thrombosis, pulmonary thromboembolism etc.

#### Dose adjustment

Any change in warfarin dose made including reduction, increase, and omission for out of the therapeutic range.

#### Illegible medical charts

Refers to patient medical charts that were recorded as undetectable INR results and unregistered test dates for the INR.

#### Prescribed medications

Warfarin was the primary anticoagulant. Concomitant medications included heparin (bridging therapy), aspirin, and various cardiovascular drugs.

#### Time in Therapeutic Range (TTR)

A measurement of the quality of anticoagulation intensity, represented as the length of time the patient’s INR was within the therapeutic range. An increased TTR is associated with bleeding and thromboembolism risk reduction and is used as a surrogate marker to assess outcomes. “Good anticoagulation quality” is declared by TTR ≥ 65%, whereas “poor” anticoagulation quality is confirmed by TTR < 65% (Liyew et al., 2021; Yimer et al., 2021).

#### Warfarin dose not adjusted

No evidence of a reduction, an increase, or omission of warfarin dose for out of the therapeutic range.

### Ethical approval and consent to participate

The study was approved by the Internal Review Board (IRB) of Wallaga University (Minutes No: 1,035/2023). All methods were carried out in accordance with relevant guidelines and regulations. A waiver for informed consent was obtained from the IRB due to the retrospective nature of the study.

## Results

### Socio-demographic characteristics of the study participants

A total of 402 patients with warfarin therapy indications were enrolled in the study. Among the study participants, 271 (67.4%) were female patients. The mean age of the study participants was 38.9 ± 17.9 years (18–80 range). Among the study participants, 307 (76.4%) had comorbid conditions, of which 74 (18.4%) had congestive heart failure (Table 1).

**TABLE 1:** Characteristics of patients on warfarin therapy at WURH and NCSH, Nekemte, Western Ethiopia from 1 April 2021-31 March 2023 (N = 402).

Characteristics		N	%
Sex	Male	131	32.6
	Female	271	67.4
Age	18–40	244	60.7
	41–64	99	24.6
	65–74	45	11.2
	≥75	14	3.5
Presence of comorbidity	Yes	307	76.4
	No	95	23.6

Types of comorbid conditions	Congestive heart failure	74	18.4
	Anemia	42	10.4
	Hypertension	42	10.4
	Hypertension and Congestive heart failure	37	9.2
	Congestive heart failure and Dilated cardiomyopathy	12	3
	Ischemic stroke	12	2.2
	Congestive heart failure and Degenerative valvular heart disease	11	2.7
	Tuberculosis	8	2
	Hypertension, Congestive heart failure, and Ischemic stroke	5	1.2
	Hypertension, Diabetes mellitus, and Acute coronary syndrom	4	1
	Hypertension and Diabetes mellitus	4	1
	Hypertension and Acute coronary syndrome	3	0.7
	Diabetes mellitus	1	0.2
	Congestive heart failure and Anemia	1	0.2
	Others^a^	51	12.7

^a^ Urinary tract infection, community-acquired pneumonia, retroviral infection, cellulitis, peptic ulcer disease, cervical cancer, endometrial cancer, prostate cancer, bronchial asthma, dyslipidemia, and ischemic heart disease.

### Types of prescribed medications

On average, 4.7 ± 2.01 (1–12 in range) medications were prescribed. The most frequently prescribed medication was heparin 326 (81.1%) followed by tramadol 257 (63.9%) (Table 2).

**TABLE 2:** Types of prescribed medications among outpatients on warfarin therapy at WURH and NCSH, Nekemte, Western Ethiopia from 1 April 2021-31 March 2023 (N = 402).

Variables		N	%
Number of prescribed medications	1–2	35	8.7
	3–4	194	48.3
	≥5	173	43
Types of prescribed medications			
Non-steroidal anti-inflammatory drugs and opioids	Diclofenac	15	3.7
	Ibuprofen	1	0.2
	Acetaminophen	42	10.4
	Tramadol	257	63.9
	Morphine	9	2.2
	Meloxicam	3	0.7
	Pethidine	1	0.2
Anticoagulants	Heparin	326	81.1
Antiplatelet agents	Aspirin	67	16.7
	Clopidogrel	4	1
Antimicrobials	Ceftriaxone	79	19.7
	Azithromycin	39	9.7
	Benzathine penicillin	34	8.5
	Cefepime	3	0.7
	Vancomycin	8	2
	Metronidazole	19	4.7
	Amoxicillin-clavulanic acid	6	1.5
	Cloxacillin	15	3.7
	Ciprofloxacin	3	0.7
	Norfloxacin	1	0.2
	Cephalexin	8	2
	Cefixime	2	0.5
	Ceftazidime	3	0.7
	Ketoconazole	1	0.2
	Nitrofurazone	1	0.2
Cardiovascular drugs	Furosemide	85	21.1
	Enalapril	76	18.9
	Metoprolol	77	19.2
	Bisoprolol	1	0.2
	Propranolol	1	0.2
	Spironolactone	5	1.2
	Digoxin	46	11.4
	Amlodipine	43	10.7
	Hydrochlorothiazide	3	0.7
Lipid-lowering agents	Atorvastatin	39	9.7
	Lovastatin	1	0.2
Endocrine drugs	Insulin	7	1.7
	Metformin	4	1
	Propyl thio uracil	4	1
Proton pump inhibitors	Omeprazole	12	3
	Pantoprazole	1	0.2
Histamine-2 receptor blockers	Cimetidine	21	5.2
	Ranitidine	3	0.7
Corticosteroids	Prednisolone	2	0.5
	Hydrocortisone	2	0.5
Other drugs^a^		113	28.1

^a^ Ferrous sulfate, folic acid, cyanocobalamin, artesunate, artemether-lumefantrine, albendazole, mebendazole, isoniazid, rifampin, pyrazinamide, ethambutol, pyridoxine, dolutegravir, lamotrigine, efavirenz, trimethoprim/sulfamethoxazole, chemotherapy, salbutamol puff, haloperidol, diazepam, diloxanide furoate, potassium chloride, vitamin K, neurobin, metoclopramide.

### Indications of warfarin therapy

Warfarin was majorly 266 (66.2%) indicated for the management of deep venous thrombosis in this study (Table 3).

**TABLE 3:** Indications of warfarin therapy among outpatients at WURH and NCSH, Nekemte, Western Ethiopia from 1 April 2021-31 March 2023 (N = 402).

Indications of warfarin therapy	N	%
Deep vein thrombosis	266	66.20
Atrial fibrillation	74	18.4
Pulmonary thromboembolism	52	12.9
Left atrial thrombus	4	1.0
Inferior vena cava thrombus	4	1.0
Acute cerebral venous thrombosis	2	0.5

### Duration of warfarin therapy use

**FIGURE 2:**
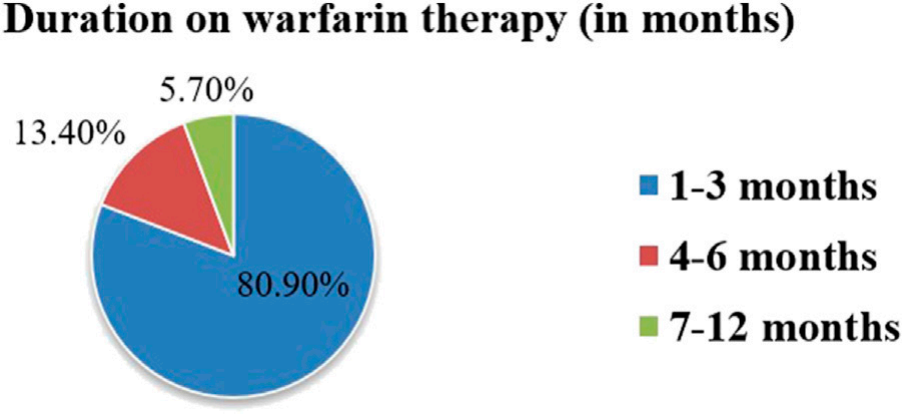
Duration of warfarin therapy use among outpatients at WURH and NCSH, Nekemte, Western Ethiopia from 1 April 2021-31 March 2023 (N = 402).

Warfarin therapy was prescribed for 1–3 months in 80.9% of study participants. This aligns with DVT/PE management protocols in the setting. The mean duration of warfarin therapy prescription was 2.2 ± 2.0 months (1–11 months in range) (Figure 2). Patients in this group had significantly lower TTR compared to those on longer therapy, reflecting the challenges of initial dose adjustments and stabilization.

### Anticoagulation quality with warfarin therapy

In the present study, the anticoagulation quality with warfarin therapy was reported to be poor (TTR <65%) in 366 (91%) of patients. Only 36 (9%) of patients had good anticoagulation quality with warfarin therapy (TTR ≥65%).

### Time in the therapeutic range

**FIGURE 3:**
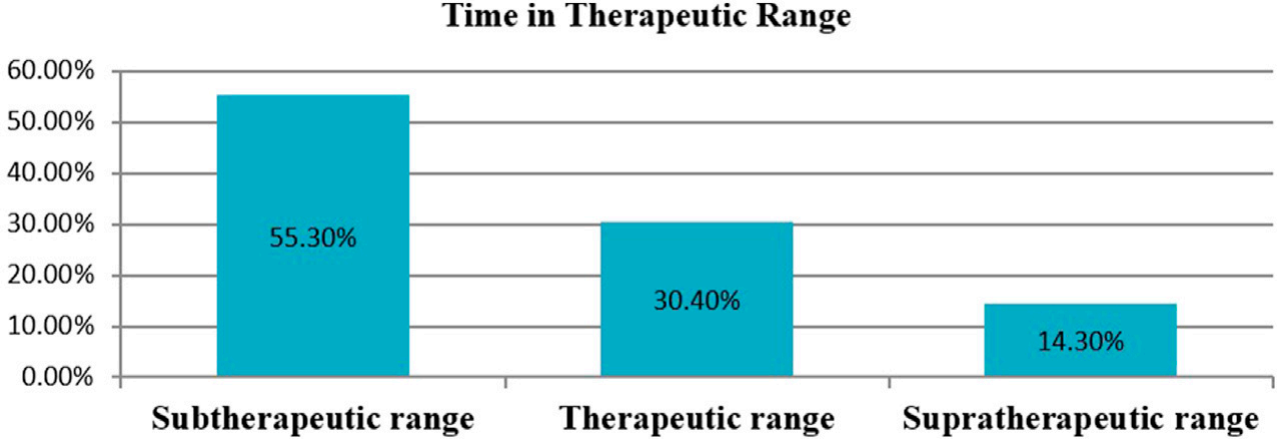
The percentage of time in therapeutic range among outpatients on warfarin therapy at WURH and NCSH, Nekemte, Western Ethiopia from April 1, 2021-March 31, 2023 (N = 402).

In this study, a total of 18,611 days of INR was tested and the mean INR measurement was 46.2 days. Time in the therapeutic range was calculated by the Rosendaal method using a developed Excel template. The mean proportion of time spent within the therapeutic range was 30.4%. The proportion of time spent in the subtherapeutic and supratherapeutic ranges was 55.3% and 14.3%, respectively (Figure 3).

### INR distribution

#### Proportion of target INR range (INR range of 2.0–3.0)

**FIGURE 4:**
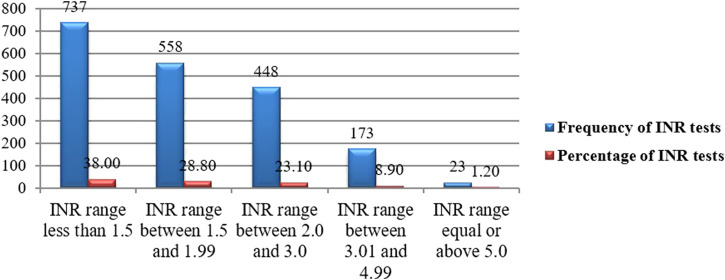
INR distribution for a target range of 2.0–3.0 among outpatients on warfarin therapy at WURH and NCSH, Nekemte, Western Ethiopia from 1 April 2021-31 March 2023 (N = 402).

The target range of INR for all patients was 2.0–3.0. A total of 1,939 INR monitoring was recorded with a mean of 4.80 ± 2.40 (2–13 INR range) and the median number of INR tests per patient was 4 (IQR: 3–6) during the study period. Among the total INR measurements, 1,295(66.8%), 448(23.1%), and 196 (10.1%) were in the subtherapeutic, therapeutic, and supratherapeutic range, respectively (Figure 4). Subtherapeutic INR was managed through warfarin dose escalation in 68% of cases. A significant proportion of DVT patients received heparins as part of initial anticoagulation management, but bridging therapy with heparin was used in 15% of patients with critically low INR and high thrombotic risk.

#### Dose adjustment of warfarin therapy

**FIGURE 5:**
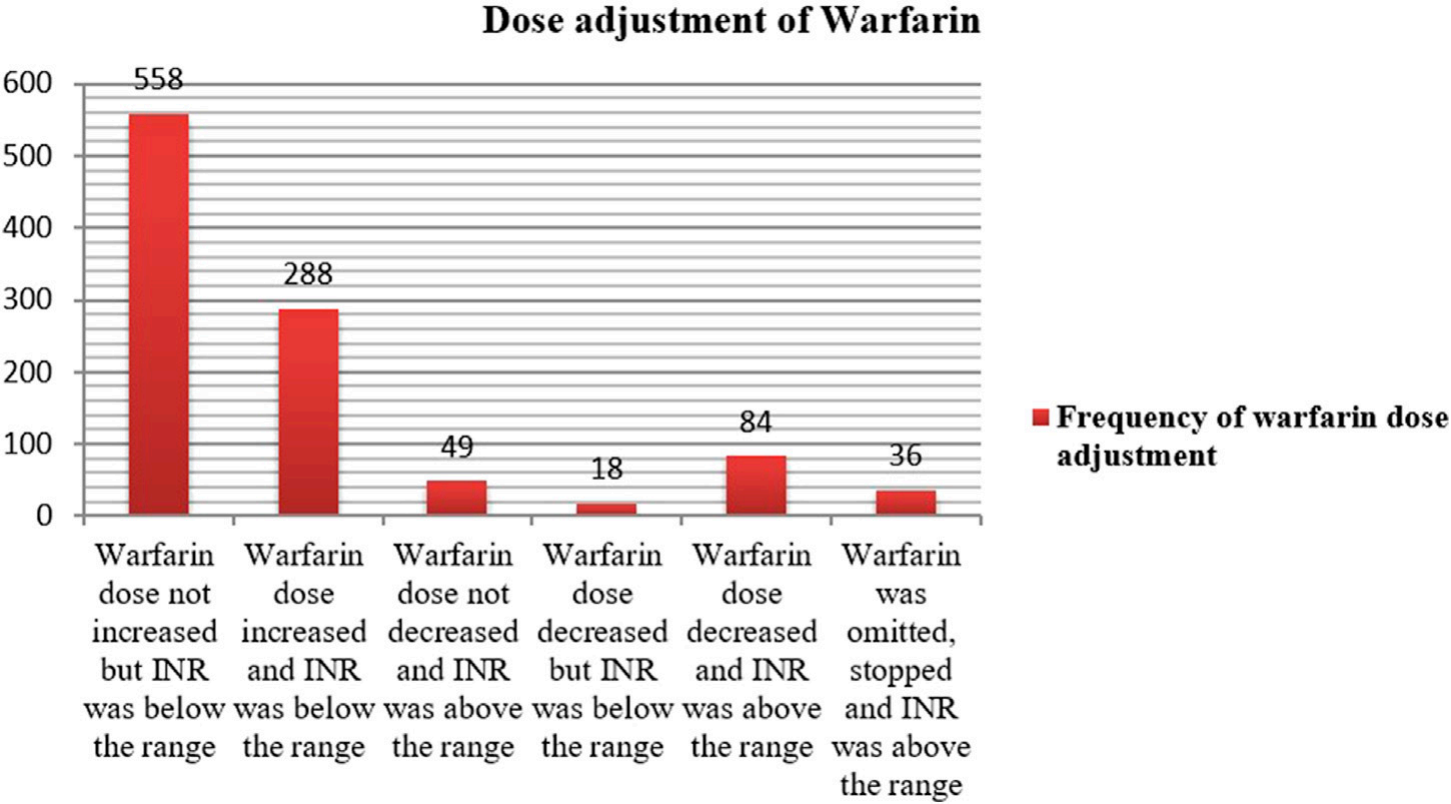
Dose adjustment status of warfarin therapy for out of the target INR range among outpatients at WURH and NCSH, Nekemte, Western Ethiopia from 1 April 2021-31 March 2023 (N = 402).

The mean daily dose of warfarin was 4.5 ± 1.9 mg (range: 2.5–10 mg). Warfarin dose was adjusted 426 times with an average of 1.73 ± 1.0 (1–5 times dose adjustment). However, the dose was not adjusted 558 times, in which the INR test was below the range. Among the total number of dose adjustments made, 288 were increased with documented INR tests below the range. Dose reduction and omission were made 84 and 36 times, respectively with evidence of INR test above the therapeutic range (Figure 5). While we could not formally define warfarin resistance, 11 patients required daily doses ≥7.5 mg, which may indicate potential resistance.

### Adverse clinical outcomes

Among the total study participants who received warfarin therapy, 19 and 32 patients experienced warfarin-related bleeding events and thromboembolic complications, respectively as adverse clinical outcomes. The majority of the patients 13 (68.4%) who experienced bleeding events bleed during the supratherapeutic range. Minor bleeding events 11 (57.9%) were the most predominant. In comparison, thromboembolic complications were observed during the subtherapeutic range in the majority 29 (90.6%) of the study participants.

### Factors associated with poor anticoagulation quality with warfarin therapy

All relevant variables were checked for statistically significant association with poor anticoagulation quality with warfarin therapy (TTR <65%) using bivariable and multivariable logistic regression analysis. A total of three variables with p-values ≤0.25 were identified first, by performing bivariable logistic regression analysis and were candidates for multivariable logistic regression analysis. In bivariable logistic regression analysis, the sex of participants, congestive heart failure, and aspirin use showed a statistically significant association. In multivariable analysis, aspirin use (AOR = 2.685 [CI: 0.872–10.277]; p-value = **0.002**) and congestive heart failure (AOR = 4.392 [CI: 1.028–18.768]; p-value = **0.046)** were 2 and 4 times more likely associated with poor anticoagulation quality with warfarin therapy, respectively (Table 4).

**TABLE 4:** Bivariable and multivariable logistic regression analysis of factors associated with poor anticoagulation quality with warfarin therapy at WURH and NCSH, Nekemte, Western Ethiopia from 1 April 2021-31 March 2023 (N = 402).

		Anticoagulation quality					
		Poor	Good	COR (95% CI)	P-value	AOR (95% CI)	P-value
Sex	Male	123	8	1		1	1
	Female	243	28	0.564 (0.250–1.275)	0.169	0.742 (0.323–1.708)	0.484
Polypharmacy	1–2 3–4 ≥5	33 171 162	2 23 11	1 0.451 (0.101–2.004) 0.893 (0.189–4.216)	1 0.295 0.886		
Duration of warfarin therapy use	1–3 4–6 7–12	295 50 21	30 4 2	1 1.271 (0.429–3.764) 1.068 (0.239–4.777)	1 0.665 0.932		
Congestive heart failure	Yes	72	2	4.163 (0.977–17.734)	0.054	4.392 (1.028–18.768)	0.046^a^
	No	294	34	1	1	1	1
Aspirin use	Yes	64	3	**2.331(0.636–9.212)**	**0.069**	**2.685(0.872–10.277)**	**0.002** ^a^
	No	302	33	1	1	1	1

AOR: adjusted odds ratio, CI: confidence interval, COR: crude odds ratio.

^a^ Bold values show independent predictors of poor anticoagulation quality with warfarin therapy.

## Discussion

This study aimed to evaluate the quality of anticoagulation, and associated factors among outpatients on warfarin therapy at public hospitals in Nekemte town. The study involved 402 outpatients who were prescribed warfarin during the study period. In our study, more than three-fourths of patients had poor anticoagulation quality with warfarin therapy.

The study revealed a total of 1,939 INR monitoring, which was lower than the previous study in Ethiopia (3162 INR) (Yimer et al., 2021). This INR monitoring gap may be the lack of a separate anticoagulation clinic in the present study. The reported mean of 4.8 ± 2.4 INR measurements per patient is indeed for the entire 1-year follow-up period. Given that most patients were treated for DVT, the frequency of INR monitoring may have been lower than expected for a chronic anticoagulation cohort. However, our study showed a higher frequency of INR monitoring compared to a previous study conducted at a private cardiac center in Ethiopia (1562 INR) (Getachew et al., 2023). The difference in INR monitoring may be due to the variation in warfarin anticoagulation control.

The warfarin dose was adjusted 426 times for non-therapeutic INR range during a 2-year follow-up, significantly lower than two studies in Ethiopia at Saint Paul Hospital Millennium Medical College (2064 times) (Yimer et al., 2021) and two private cardiac centers (1764 times) (Getachew et al., 2023). The discrepancy may be attributed to differences in the patient’s socio-demographic and clinical characteristics. Our study found a mean TTR of 30.4%, similar to Pakistan (34.9%) (Hakem et al., 2018) and Ethiopia at Tikur Anbessa Specialized Hospital (29%) but lower than the European Cardiac Society guidelines (≥70%) (Task et al., 2020), South Africa (47%) (Ebrahim et al., 2018), Malaysia (47%) (Leh et al., 2020) and Ethiopia at Saint Paul Hospital Millennium Medical College (42.03%) (Yimer et al., 2021) and two private cardiac center (47.24%) (Getachew et al., 2023).

Our study also demonstrated that achieving an optimal TTR remains a challenge among patients on warfarin therapy. The mean TTR observed in our study was lower than that reported in studies from western countries, where TTR often exceeds 60% (Jones et al., 2022). The suboptimal TTR may be attributed to factors such as inadequate monitoring, medication adherence issues, and co-administration of interacting drugs. Moreover, infrequent INR measurements can bias TTR estimates when using Rosendaal’s method.

Patients with good anticoagulation quality with warfarin therapy were lower in this study (9%) compared to studies from Poland (Sawicka-Powierza et al., 2018), Pakistan (Hakem et al., 2018), and Botswana (Mwita et al., 2018), where 54.7%, 30.8%, and 22.8% of patients had good anticoagulation quality with warfarin therapy, respectively. The probable reason for this discrepancy might be linked to the concomitant use of multiple drugs which negatively affect TTR. Moreover, the reason may be due to lack of establishing anticoagulation clinics, pharmacist-led warfarin management, and patient education. However, the finding is comparable to a study conducted in Ethiopia (Yimer et al., 2021), where 12.67% of patients on warfarin therapy had good anticoagulation quality.

This study found a significant association between aspirin use and poor anticoagulation quality in a multivariable logistic regression analysis (AOR = 2.685 [CI: 0.872–10.277]; p-value = 0.002). This is supported by the study conducted in the United Kingdom, where chronic use of pain medications, such as paracetamol, NSAIDs, or opioids, increases the risk of poor anticoagulation control (Filipa et al., 2015). Similarly, a study from Australia (Boyce et al., 2018) found that patients on aspirin had poor warfarin control, resulting in an increased risk of minor bleeding (p = 0.048) (Boyce et al., 2018). While aspirin can enhance the antithrombotic effect of warfarin, it also increases the risk of bleeding by inhibiting platelet aggregation, independent of INR levels (Garcia and Crowther, 2019). This finding underscores the necessity of balancing the benefits and risks when prescribing dual antithrombotic therapy.

Congestive heart failure was another independent predictor of poor anticoagulation quality with warfarin therapy (AOR = 4.392; [CI: 1.028–18.768]; p-value = 0.046). Consistently, the study from the United States (Nelson et al., 2013) and Ethiopia (Yimer et al., 2021), found that congestive heart failure increases the risk of poor warfarin anticoagulation quality (AOR = 1.41; [CI 1.28–1.56]; p < 0.001) and (AOR = 2.467; [CI 1.014–6.005]; p = 0.047), respectively. Furthermore, heart failure, a common comorbidity in our population, has been associated with reduced warfarin metabolism and fluctuations in INR levels (Rodriguez et al., 2020). A study from Poland revealed that arterial hypertension (OR = 2.74; [1.06–7.10]; p-value = 0.038), amiodarone therapy (OR = 4.22; [1.30–13.70]; p-value = 0.017), and body mass index (kg/m^2^) (OR = 1.11; [1.02–1.21]; p-value = 0.013), are associated with poor anticoagulation quality (Ciurus et al., 2015). These findings differ from the present study due to differences in age, the absence of amiodarone indications, and a lack of weight and height data. Amiodarone is a well-known inhibitor of warfarin metabolism via cytochrome P450 enzymes (Garcia and Crowther, 2019), often leading to increased INR levels and a higher risk of supratherapeutic anticoagulation. However, in our study, amiodarone use was not associated with a higher likelihood of subtherapeutic INR. This discrepancy may be due to the fact that amiodarone was not frequently co-prescribed with warfarin in our study population. These factors highlight the need for individualized dosing and closer monitoring in patients receiving amiodarone. Amiodarone is indeed known to inhibit warfarin metabolism and increase INR levels, our analysis not identified its association with a higher likelihood of subtherapeutic TTR in our study. The reason might be amiodarone was not prescribed with warfarin in the present study. Our study found that no significant association between anticoagulation quality and socio-demographic characteristics such as age and gender. The younger age distribution of our sample might influence the generalizability of our findings, particularly when comparing to previous studies that primarily focused on older populations using warfarin. We noted that although not statistically significant, female patients had a lower likelihood of good TTR. Cultural factors, healthcare access, and potential differences in adherence may explain this trend.

Regarding adverse clinical outcomes, our recalculated data confirmed that among 19 patients with bleeding events, 13 (68.4%) were in the supratherapeutic INR range. This reinforces the well-established correlation between high INR levels and increased bleeding risk (Patel et al., 2023).

## Limitations of the study

The present study is not without limitations. For retrospective nature of the present study, data on influence of clinical factors such as smoking status and body mass index (BMI) were not consistently recorded in patient charts, which limited our ability to analyze these factors. The younger sample may limit the direct applicability of our results to older patients, who typically exhibit different pharmacokinetic and pharmacodynamic responses to warfarin, as well as a higher burden of comorbidities and polypharmacy. Moreover, the short duration of follow-up may have led to an underestimation of TTR, as patients with extended therapy generally achieve better INR control over time. When INR values are recorded at relatively long intervals, Rosendaal’s interpolation method may introduce inaccuracies by assuming a linear trend between measurements, potentially misrepresenting fluctuations in anticoagulation control. INR testing was primarily performed using laboratory-based blood testing; point-of-care devices were not available. Atrial fibrillation had no separate registration code; this might lead to underestimation. The absence of patients with mechanical valve replacement likely reflects the limited availability of valve replacement surgeries in the study area. Laboratory parameters that affect INR, such as baseline creatinine levels and liver enzymes were not routinely documented in medical records.

## Conclusion

Our study revealed that the anticoagulation quality with warfarin therapy was poor. The majority of the patients spent their time out of the INR target range. Factors identified to be significantly associated with poor anticoagulation quality with warfarin therapy were the use of aspirin and congestive heart failure.

## Recommendations

Separate anti-coagulation clinics should be established in the hospitals to achieve better anticoagulation control. Clinical pharmacist’s role in achieving good anticoagulation control has become crucial worldwide; therefore, there needs careful emphasis on the best patient care in the country. This study was retrospective; we recommend future prospective studies to include adherence evaluation and should incorporate socio-economic status variable.

## Data Availability

The raw data supporting the conclusions of this article will be made available by the authors, without undue reservation.

## Abbreviations

AF: Atrial fibrillation. AOR: Adjusted odds ratio. CI: Confidence interval. COR: Crude odds ratio. DDI: Drug-drug interaction. INR: International normalized ratio. MHV: Mechanical Heart Valve. NCSH: Nekemte Comprehensive Specialized Hospital. TTR: Time in the therapeutic range. VTE: Venous thromboembolism. WURH: Wallaga University Referral Hospital.
